# New insights into different adipokines in linking the pathophysiology of obesity and psoriasis

**DOI:** 10.1186/s12944-019-1115-3

**Published:** 2019-09-14

**Authors:** Yi Kong, Suhan Zhang, Ruifang Wu, Xin Su, Daoquan Peng, Ming Zhao, Yuwen Su

**Affiliations:** 1 Department of Dermatology, Hunan Key Laboratory of Medical Epigenomics, the Second Xiangya Hospital of Central South University, 139 Renmin Middle Road, Changsha, 410011 Hunan China; 2 Department of Cardiovascular Medicine, the Second Xiangya Hospital of Central South University, Changsha, Hunan China

**Keywords:** Adipokine, Psoriasis, Obesity, Pathophysiology, Treatment

## Abstract

Psoriasis is a chronic, systemic, hyper-proliferative immune-mediated inflammatory skin disease. The results of epidemiological investigations have shown that psoriasis affects around 2% of the general population worldwide, and the total number of psoriasis patients is more than 6 million in China. Apart from the skin manifestations, psoriasis has been verified to associate with several metabolic comorbidities, such as insulin resistance, diabetes and obesity. However, the underlying mechanism is still not elucidated. Adipocytes, considered as the active endocrine cells, are dysfunctional in obesity which displays increased synthesis and secretion of adipokines with other modified metabolic properties. Currently, growing evidence has pointed to the central role of adipokines in adipose tissue and the immune system, providing new insights into the effect of adipokines in linking the pathophysiology of obesity and psoriasis. In this review, we summarize the current understanding of the pathological role of adipokines and the potential mechanisms whereby different adipokines link obesity and psoriasis. Furthermore, we also provide evidence which identifies a potential therapeutic target aiming at adipokines for the management of these two diseases.

## Introduction

Psoriasis is a chronic, systemic, hyper-proliferative, immune-mediated inflammatory skin disease. The typical psoriatic skin lesions present as silver-whitish scales with sharply demarcated, red and thickened areas [1]. The results of epidemiological surveys have shown that psoriasis affects around 2% of the general population worldwide, and the total number of psoriasis patients is more than 6 million in China [2, 3]. Obesity, defined as having a body mass index (BMI) greater than 30 kg/m^2^, is associated with a series of health problems that are always grouped together as metabolic syndromes [4]. Of note, obesity has nearly doubled during the past four decades all over the world [5]. In China, the prevalence of overweight was 25.8% (25.9% in males and 25.7% in females), while that of obesity was 8.1% (8.4% in males and 7.6% in females) in 2014 [6], posing serious risks to the future health and reduce the quality of life in the general population.

Presently, questions concerning the relationship of psoriasis and obesity have focused on the potential biological basis underlying the development of these two diseases. Multiple studies assume that adipokines, a type of cytokine synthesized and secreted by adipocytes, play an important role in linking the pathological process of psoriasis and obesity. As shown previously, adipocytes are the predominant cell type in adipose tissue, which are not only a passive container for storing excess energy in the form of fat [7] but also an important source of hormones and endocrine molecules, such as adipokines [8–10]. Under the obese status, adipocytes are enlarged and dysfunctional [11], secreting increased quantities of adipokines and exhibiting other modified metabolic properties [12]. Some adipokines, including hormones, cytokines and other proteins [13–17], possess pro-inflammatory properties that involve in the pathogenesis of inflammatory diseases, such as asthma [18, 19], rheumatoid arthritis (RA) [20–23] and psoriasis [12, 24, 25]. Concerning this notion, studies have demonstrated a positive relationship between psoriasis and several metabolic comorbidities, including obesity, hypertension and dyslipidemia [25, 26]. Indeed, the prevalence of obesity in psoriatic patients is higher than that in the healthy population [27], nonetheless, the underlying mechanism is still not elucidated. In this review, we summarize the current understanding of the pathological role of adipokines and the potential mechanisms whereby different adipokines link obesity and psoriasis. Furthermore, we also provide evidence which identifies a potential therapeutic target aiming at adipokines for the management of these two diseases.

## Relationship between obesity and psoriasis focusing on the prevalence rate and pathology

It has been known since the early twenty-first century that psoriasis has a greater tendency to associate with obesity and its related metabolic syndromes, a finding which has been replicated in many studies from around the world. Early in 2006, Neimann et al. have already determined a positive correlation between the increased BMI and the extent of psoriasis severity measured by the psoriasis area and severity index (PASI) score [28]. Similar result was presented by a meta-analysis in which the average prevalence of obesity in psoriasis patients was about 23.5%; concurrently, patients with high PASI scores exhibited a higher prevalence rate of obesity [29]. A systematic review including 25 studies found a higher prevalence rate of dyslipidemia in psoriasis patients with higher PASI scores [30]. The results of another comprehensive study also showed a significant relationship between PASI scores and dyslipidemia, with adjusted ORs of 1.22, 1.56, and 1.98 for mild, moderate, and severe psoriasis, respectively [31]. Since dyslipidemia is one of the hallmarks of obesity, we can infer from these results that dyslipidemia might contribute to the risk of psoriasis by promoting the pathological processes that lead to obesity. On the other hand, the incidence of psoriasis in obese children has begun to gain appreciation. Compelling evidence has demonstrated that the pediatric psoriasis is associated with a risk of pediatric metabolic syndromes [32–35], indicating an association between psoriasis and obesity in children and emphasizing the importance of careful assessment of metabolic comorbidities in psoriatic youngsters.

The association between obesity and psoriasis has also been confirmed in animal studies. Using an obese mice model with psoriasiform dermatitis induced by imiquimod (IMQ), Kanemaru et al. found that the obese status could acutely exaggerate the severity of psoriasiform dermatitis in mice. Meanwhile, the mice exhibited higher serum levels of psoriasis mediators, such as interleukin-22 (IL-22), IL-17A and its downstream molecule regenerating islet-derived 3γ (Reg3γ), which has been confirmed to be a critical molecule in psoriatic epidermal hyperplasia [36]. The results suggest that the obese status can exacerbate psoriasis-form dermatitis, at least partly, by upregulating the pro-inflammatory factors.

In summary, obesity can promote the severity of psoriasis; concurrently, psoriasis also occurs more frequently in obese people. However, the underlying mechanisms which link psoriasis with obesity has not yet been clarified. Both psoriasis and obese status could facilitate the metabolic alterations that could be the primary and triggering events [37, 38]. Nevertheless, both aberrant conditions could also develop independently because of the shared risk factors, such as genetics or lifestyles.

## Adipokines are pathogenic factors which link psoriasis and obesity

**Fig. 1:**
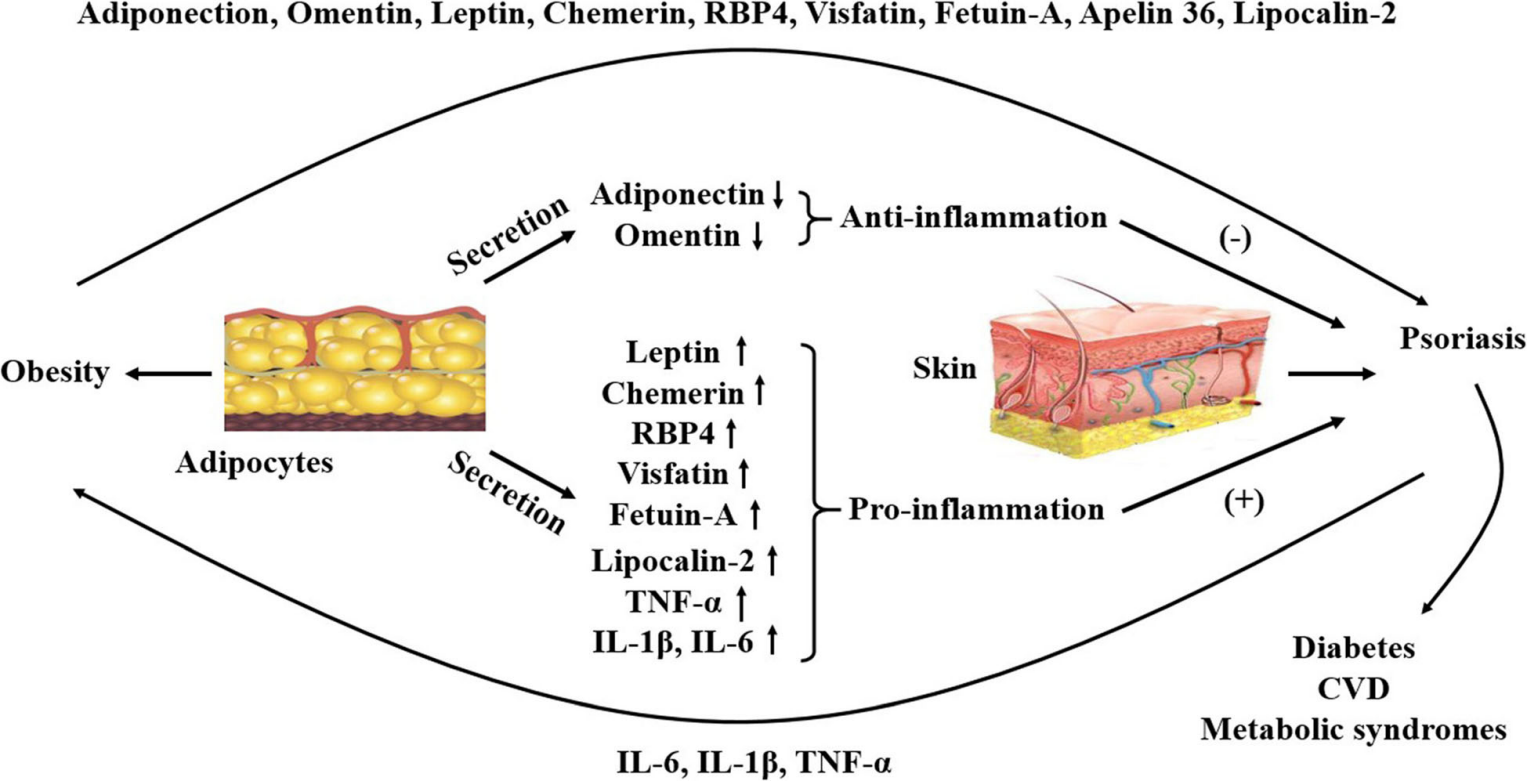
Psoriasis-signature cytokines, such as TNF-α, IL-1β and IL-6, have effects on adipose tissue being involved in key mechanisms of TG metabolism and differentiation of pre-adipocytes, including increased risk of obesity. Secreted adipokines, such as leptin, chemerin, RBP4, visfatin, fetuin-A, apelin 36 and lipocalin-2, could amplify the immune response and promote immune-mediated diseases by their pro-inflammatory effects; however, adiponectin and omentin shows anti-inflammatory effects, and the levels of adiponectin and omentin obviously decrease in obese patients. The figure briefly present the function of different adipokines in linking psoriasis and obesity

Under the obese status, the pro-inflammatory adipokines are excessively synthesized and secreted by dysfunctional adipocytes. However, the anti-inflammatory adipokines are produced with a less extent. Of note, these inflammatory includes hormones, cytokines and other proteins. Given the vast array of inflammatory conditions commonly seen in obesity and psoriasis, many of which pose a great burden to the individual and society, it is important to seek a better fundamental understanding of those adipokines. Furthermore, since psoriasis and obesity always risk factors, we hypothesize that the mechanism of linking obesity and psoriasis could be explained by adipokines. A summary of the reported data about the characteristics of different adipokines in obesity and psoriasis is presented in Table 1 and Fig. 1.

**Table 1:** The important adipokines and the possible effects on obesity and psoriasis

Adipokine	Basic role of adipokine	Roles and effects on psoriasis	Roles and effects on obesity
Adiponectin	Anti-inflammatory Anti-atherogenic	Inversely correlates with psoriasis severity, especially the HMW subtype An increase in serum levels of adiponectin in psoriatic patients Suppresses inflammation and immune responses	Decreased in obesity Protects against obesity-linked metabolic dysfunction in mouse models A therapy associated with metabolic syndromes Possesses anti-inflammatory properties by inhibiting the NF-κB signaling pathway
Leptin	Pro-inflammatory Regulates expression of adhesion molecules and angiogenesis	Increased in psoriasis Increases Th-1 lymphocytes and the Th-1 type cytokines Decreases Th-2 type cytokines Promotes the secretion of several pro-inflammatory factors Regulates proliferation of keratinocytes	Positively correlated with BMI Regulates feeding behavior through the central nervous system
Chemerin	Pro-inflammatory Chemotactic protein Released mainly by adipocytes and dermal fibroblasts	Involved in the recruitment of pDC in the early stage of psoriasis Binds to both the signaling and non-signaling receptors Promotes the pDC transmigration	Positively correlated with BMI Considered as a biomarker in the development of obesity Promotes the adipogenesis differentiation of pre-adipocytes
Visfatin	Pro-inflammatory Binds to insulin receptors Activates T lymphocytes in the immune system	Enhances production of antimicrobial peptides in human keratinocytes The gene of visfatin is upregulated in psoriatic patients	Positively correlated with abdominal obesity Causes dyslipidemia Negatively correlated with the plasma level of HDL-C Promotes the secretion of VEGF Inhibits the expression of metalloproteinases
Omentin	Anti-inflammatory Induces the expression and phosphorylation of NOS Stimulates the vasodilation of blood vessels	Lower levels in psoriatic patients compared to the healthy controls Increased after treatment of psoriasis Attenuate the TNF-α-induced adhesion molecule expression and monocyte adhesion	Increases insulin sensitivity in human adipocytes Risk factor for insulin resistance Omentin-1 is positively correlated with plasma level of adiponectin and is inversely correlated with BMI or WHR
TNF-α	Pro-inflammatory Promotes the development of metabolic syndrome and vascular diseases	Increased in psoriatic patients Positively correlated with PASI Facilitates production of pro-inflammatory cytokines synthesized by T lymphocytes and macrophages	Increased in obese patients Positively correlated with BMI Induces dyslipidemia
IL-1β	Pro-inflammatory Promotes the development of metabolic syndrome and vascular diseases	Increased in psoriatic patients Positively correlated with PASI Activates proliferation of keratinocytes	Increased in obese patients Positively correlated with BMI Induces dyslipidemia Promotes the inflammation-induced destruction of pancreatic β-cells
IL-6	Pro-inflammatory Impairs the insulin release	Increased in psoriatic patients Positively correlated with PASI Activates T lymphocytes and the proliferation of keratinocytes	Increased in obese patients Positively correlated with BMI Induces dyslipidemia
RBP4	Secreted by adipocytes, macrophages and hepatocytes Transfers retinol (Vitamin A)	Decreased in psoriatic patients Positively correlated with the PASI score Positively correlated with the circulating levels of inflammatory factors Reduces after the treatment with acitretin	Produced by visceral adipocytes under the obese status Plays a major role in insulin resistance Positively associated with BMI, WHR, plasma level of TG and systolic blood pressure
FetA	Produced by adipocytes, keratinocytes and hepatocytes Inhibit the lipid efflux especially within macrophages	Induces the synthesis and secretion of pro-inflammatory adipokines Promotes transformation of the anti-inflammatory M2-phenotype macrophages into the pro-inflammatory M1-phenotype macrophages Increased in psoriatic patients Positively correlated with PASI scores	FFA could enhance the production of FetA in hepatocytes and adipocytes Reduces after treatment of weight loss in obese patients
LCN2	Expressed in human livers, lungs, kidneys and adipose tissues A component of the innate immune system Functions in the acute phase response to infection Induces apoptosis Involves in several inflammatory diseases	Increased in psoriatic patients Positively correlated with PASI scores Up-regulates in keratinocytes of psoriatic skin lesions Positively correlated with IL-1β	Increased in obese patients Positively correlated with BMI Induces dyslipidemia

*Abbreviations*: *HMW* high molecular weight, *NF-κB* nuclear factor kappa B, *BMI* body mass index, *pDC* plasmacytoid dendritic cells, *HDL-C* high density lipoprotein cholesterol, *VEGF* vascular endothelial growth factors, *NOS* nitric oxide synthase, *TNF-α* tumor necrosis factor-alpha, *WHR* waist-hip ratio, *PASI* psoriasis area and severity index, *RBP4* retinol binding protein 4, *FetA* fetuin-A, *LCN2* lipocalin-2, *IL-6* interleukin-6, *FFA* free fatty acid

### Adiponectin

Adiponectin is exclusively synthesized by adipocytes [39], which has been shown to enhance insulin sensitivity and fatty acid oxidation. It is worth noting that adiponectin could also prevent atherosclerosis and improve anti-inflammation, playing a protective role in the pathogenesis of metabolic syndromes. In obese humans and animals, the serum levels of adiponectin are decreased and negatively correlated with the levels of tumor necrosis factor-α (TNF-α) and interleukin-6 (IL-6) [40]. Additionally, adiponectin is verified to increase the production of nitric oxide (NO) in endothelial cells by activating the phosphatidylinositol-3 kinase/Akt (PI3K/Akt) signaling pathway, thus, the lower adiponectin levels are considered to be a risk factor for endothelial dysfunction [41]. Recently, a series of studies have revealed that the adiponectin levels are reduced in patients with psoriasis and are negatively correlated with BMI [42–44]. In particular, the high molecular weight (HMW, oligomeric) form, one of the three major complexes of adiponectin, is confirmed as the most sensitive marker for obesity and psoriasis [45] and is significantly lower in psoriasis patients [46]. After the treatment of weight loss, the levels of adiponectin and the psoriasis-special alterations of skin have been improved to some extent [42].

Interestingly, the adiponectin also possesses anti-inflammatory properties by inhibiting the nuclear factor kappa-B (NF-κB) signaling pathway which consequently upregulates the secretion of IL-10 and regulates the toll-like receptors (TLRs) [47]. The mechanism whereby adiponectin affects obesity and psoriasis is potentially due to the regulatory role of adiponectin in skin inflammation, especially in IL-17-related psoriasis-form dermatitis. Consistent with this hypothesis, the adiponectin-deficient mice exhibit severe psoriasis-form skin inflammation with enhanced infiltration of IL-17-producing dermal Vγ4 + γδT cells, revealing that adiponectin could directly act on dermal γδ-T cells to suppress IL-17 synthesis [48]. Moreover, the synthesis and secretion of IL-17 by human CD4(+) or CD8(+) T cells are also inhibited by adiponectin [49]. With further research, the adiponectin is shown to significantly upregulate the expression level of sirtuin-1 (SIRT1) and peroxisome proliferator-activated receptor γ (PPARγ), which have a vital role in promoting the adipogenesis of adipocytes [50]. To date, the expression level of retinoid-related orphan receptor-γt (ROR-γt), one of the key transcription factors during the differentiation of Th17 cell, is synchronously inhibited by adiponectin [50]. These results have systematically uncovered the role of adiponectin in inhibiting adipogenesis of adipocytes and the Th17 cell-mediated inflammation, suggesting a novel mechanism which underlies the relationship between psoriasis and obesity.

### Leptin

Leptin, a hormone that is predominately produced by adipocytes in white adipose tissue [51], has been proved as a regulator of whole-body energy homeostasis through decreasing food intake and increasing energy expenditure [52]. The plasma level of leptin is elevated in both obese and psoriatic individuals, and the elevated concentration is positively correlated with BMI and PASI scores, suggesting an important role of leptin in linking psoriasis and obesity. Indeed, several meta-analyses have evaluated the circulating concentrations of important adipokines and found that the leptin concentrations are significantly higher in non-obese patients with psoriasis [53, 54], either under the fasting or the postprandial status [55, 56], indicating that the increase leptin levels in psoriatic patients might not only originate from adipocytes but also from keratinocytes and endothelial cells [57].

Accordingly, we speculate that leptin may act as a combined bridge between psoriasis and obesity through inflammatory processes. Actually, it is worth noting that the leptin-deficient mice with IMQ-induced psoriasis presented an attenuated extent of several manifestations of inflammation, such as the clinical signs of erythema, infiltration and scales in dorsal skin and ear skin [58]; however, after the pharmacological stimulation of leptin, the authors observed that the T lymphocytes isolated from those mice are more likely to be polarized to Th1 lymphocytes with an increase secretion of several pro-inflammatory factors, including IL-6, IL-8 (CXCL8) and TNF-α [59, 60]. In addition, the proliferation of keratinocyte is also enhanced during this process. These data strongly revealed that leptin plays an important role in linking the pathogenesis of psoriasis and obesity by promoting the production of pro-inflammatory mediators.

### Chemerin

Chemerin is a newly discovered adipokine which involves in the pathogenesis of inflammation, adipogenesis, angiogenesis and dyslipidemia [61]. Adipocytes, endothelial cells and skin keratinocytes have been shown to produce chemerin under the physiological status. Currently, chemerin has been considered as not only a classical chemokine but also a novel adipokine. Firstly, as a type of chemokine, chemerin mainly exhibits the chemotactic characteristics via being several cellular receptors. Previous studies have described that chemerin could bind to both the signaling and non-signaling receptors, such as G protein coupled receptor 1 (GPR1) and chemokine CC-motif receptor-like 2 (CCRL2). As known, CCRL2, which is principally expressed by keratinocytes, could promote the cellular binding capacity of chemerin and support the dendritic cells transmigration [62, 63]. On the other hand, as a vital adipokine, chemerin is shown to up-regulate in obese mammals [61, 64], and the plasma levels of chemerin are positively correlated with BMI and obesity-related biomarkers [41], pointing to a modulatory role of chemerin in the pathophysiology of obesity. Indeed, studies have demonstrated that the expression level of chemerin dramatically increases during the cellular differentiation of pre-adipocytes, and the increased chemerin could in turn stimulate the adipogenesis differentiation, leading to hyperplasia and hypertrophy of mature adipocytes [65–67].

Consistently, Coban et al. demonstrated a positive correlation between chemerin level with the PASI scores and BMI [68], and Gao et al. also found that the level of chemerin in psoriatic patients was higher than that in the general population [69]. Further, the adipose tissue isolated from obese and psoriatic patients is shown to secrete higher levels of chemerin [70]. After anti-psoriatic therapy, such as cyclosporine A, methotrexate and TNF-α blockers, the patients presented a relatively lower plasma levels of chemerin [71].

As mentioned above, chemerin potentially has the functions within keratinocytes and adipocytes which could induce an inflammatory response in psoriatic epidermis and adipose tissue. To validate this hypothesis, Wang et al. used the HaCaT cells and the primary human keratinocytes which were treated with chemerin previously and showed that chemerin could facilitate the secretion of inflammatory factors, including IL-1β, IL-8, TNF-α, and subsequently activate the NF-κB signaling pathway through the chemerin receptors [72]. Meanwhile, chemerin significantly reduced the expression level and constrained the deacetylase activity of SIRT1 through augmentation of reactive oxygen species (ROS) production. Similar results were observed by using the IMQ-induced psoriatic mice model [72]. Alternatively, in psoriasis dermis, chemerin is mainly secreted by fibroblasts, which could induce the migration of plasmacytoid dendritic cells (pDC) and the phosphorylation of the extracellular regulated protein kinases 1 and 2 (ERK1/2) in vitro [73]. Therefore, chemerin could act as a chemokine that recruits pDC to pre-psoriatic skin by binding to its cognate receptor, namely chemR23, expressed on pDC [74]. In conclusion, chemerin can promote NF-κB activation through inhibiting of SIRT1 activity by ROS production and consequently induce an inflammatory response, leading to the development of obesity and psoriasis.

### Visfatin

Visfatin, highly expressed in visceral tissues, is a type of pro-inflammatory cytokine which could upregulate the production of pro-inflammatory factors in monocytes and then increase the activation of T lymphocytes [45]. Of note, the visfatin-knockout monocytes isolated from the arthritis mice induced by collagen exhibited a reduced secretion of IL-6 and the attenuated extent of differentiation process of CD4(+) T lymphocytes into Th17 lymphocytes [75]. Furthermore, the expression of visfatin within endothelial cells could also promote the secretion of vascular endothelial growth factors (VEGF) and synchronously lead to a decrease in the expression of metalloproteinases, which caused the proliferation of endothelial cells and the formation of capillary cavities [76].

Accumulating evidence has demonstrated that visfatin is positively correlated with abdominal obesity and is negatively correlated with the plasma level of HDL-C [77]. On the other hand, an independent study of 40 psoriatic patients showed that the plasma level of visfatin was higher than that in healthy control individuals [78]. Further research has also determined that visfatin could act on the keratinocytes and amplify the inflammatory status through NF-κB and STAT3 signaling pathways and the upregulation of several chemokines gene expression, such as CXCL8, CXCL10, CCL20 and the antimicrobial peptides including cyclic adenosine monophosphate (CAMP) and S100A7 [75, 79], thus enhancing the severity of psoriasis. This effect of visfatin has also been observed by using the IMQ-induced psoriatic mice model, in which the antimicrobial peptides were enhanced by the treatment of visfatin. Given that the antimicrobial peptides can activate the functions of pDC which may induce the development of inflammation, these observations shed light on the potential role of visfatin in linking obesity and psoriasis [80].

### Omentin

Omentin has been shown to act on several different cells in mammals. For instance, in endothelial cells, omentin induces the expression and phosphorylation of nitric oxide synthase (NOS), which afterwards stimulates the vasodilation of blood vessels and the ischemia-induced tissue re-vascularization via endothelial NOS-dependent mechanisms [81]. In addition, omentin is verified to have a role in vascular smooth muscle cells which could attenuate the TNF-α-induced adhesion molecule expression and monocyte adhesion [82]. It should be further noted that apart from its protective function against insulin resistance, inflammation and vascular dysfunction, omentin is also suggested to function as a lectin which could bind to the galactofuranosyl residues that located on the cell walls of various bacteria [83, 84].

There are two isoforms of omentin in human circulation, namely omentin-1 and omentin-2. As demonstrated previously, these two isoforms are primarily produced by the omental adipose tissue and the epicardial adipose tissue but not the subcutaneous adipose tissue [85]. Of note, omentin, especially the isoform 1, is positively correlated with plasma level of adiponectin and is inversely correlated with BMI or waist-hip ratio (WHR), signifying an modulatory role in pathogenesis of obesity [86]. In a prospective study, the decreased omentin levels were shown to associate with an increased risk of obesity and insulin resistance [83]; other independent studies have further shown that serum omentin levels were clearly decreased and inversely correlated with PASI scores in psoriatic patients compared to the healthy control participants [87–89]. Consistently, omentin could also enhance the insulin signaling pathway and the expression of anti-inflammatory factors in adipocytes, thus suppressing the expression of different adhesion molecules induced by TNF-α [90]. To date, there are limited studies investigating omentin and its role in the pathogenesis and the clinical outcomes of psoriasis. Further studies focusing on the mechanisms whereby omentin links psoriasis and obesity are still needed.

### Cytokines

As mentioned above, under the obese status, adipocytes are dysfunctional with the excessive secretion of several pro-inflammatory cytokines, such as TNF-α, IL-1β and IL-6. Given these cytokines also commonly seen in psoriasis, which pose a great burden to the development of those two diseases, it is important to seek a fundamental understanding of these cytokines. Indeed, the role of some important cytokines in the pathogenesis of disease has been investigated.

Firstly, TNF-α, as a key regulator in inflammation which is crucial for the proliferation of T lymphocytes and keratinocytes within psoriatic lesions [91], is shown to facilitate the production of pro-inflammatory cytokines by T lymphocytes and macrophages. Some researchers found that the serum levels of TNF-α increased in psoriatic patients which was also positively correlated with PASI scores [92–95]. Additionally, a positive association between TNF-α and BMI in psoriasis was demonstrated [96]. Secondly, IL-1β is another vital pro-inflammatory cytokine that promotes psoriasis and metabolic syndromes potential via the activation of keratinocytes and the inflammation-induced destruction of pancreatic β-cells, respectively [97]. Notably, several studies have found that the IL-1β level was higher in the active phase of psoriasis and reduced in psoriatic patients after treatment consequently [98, 99]; meanwhile, a positive correlation between the serum levels of IL-1β with PASI has also been reported in psoriatic patients before and after treatment [100–102], signifying that IL-1β is an important mediator in the initiation and maintenance of psoriatic plaques. Thirdly, similar to IL-1β, IL-6 could clearly impair the insulin release and induce the production of inflammatory cytokines in adipocytes [97]. Moreover, IL-6 also mediates the activation of T lymphocytes and the proliferation of keratinocytes [102]. Current results have revealed that IL-6 is increased under the psoriatic status and positively correlates with PASI scores, at least in the more severe form of psoriasis [103, 104].

In conclusion, TNF-α, IL-6 and IL-1β are increased in psoriasis and correlate with PASI scores and obesity, leading to worsening of psoriatic lesions. The increasing results of functional analyses focusing on the cytokines could point out the potential mechanisms by which cytokines may increase susceptibility to obesity and psoriasis. However, there are also multiple cytokines which aberrantly produced under the diseases status, further studies is still needed to shed light on the physiological role of the other cytokines in linking obesity and psoriasis.

### Other important adipokines

Several other important adipokines have recently been considered as mediators of obesity and psoriasis, although there is a lack of experimental evidence that directly supports specific mechanisms. These adipokines include retinol binding protein 4 (RBP4), fetuin-A (FetA) and lipocalin-2 (LCN2).

#### RBP4

RBP4, an adipokine which predominantly secreted by adipocytes, macrophages and hepatocytes, was first discovered in 2005 [105, 106]. Especially, RBP4 is produced by the visceral adipocytes under the status of obesity and insulin resistance. It has been known since the early twenty-first century that RBP4 has a greater tendency to involve in several metabolic processes in humans and mice, a finding which has been replicated in many studies from around the world. Currently, the role of RBP4 in linking obesity and psoriasis has been given substantial attention.

Accordingly, accumulating evidence has revealed that increased RBP4 levels are positively associated with BMI, WHR, plasma level of TG and systolic blood pressure. By contrast, the serum levels of RBP4 are shown to inversely associate with plasma HDL-C levels, pointing out that RBP4 has an essential role in promoting the pathological process of obesity [107–109]. Furthermore, in psoriasis patients with obesity, the plasma levels of RBP4 are positively correlated with the PASI score and higher than those in patients with simple psoriasis [110–112]. Further research has also determined that RBP4 is positively associated with the circulating levels of inflammatory factors, such as IL-6 and TNF-α [112]. Of note, treatment with acitretin could significantly reduce the plasma levels RBP4 in psoriasis patients [113], suggesting that RBP4 is a potential target for treatment of psoriasis.

#### FetA

FetA, encoded by FETA genes, is produced by adipocytes, keratinocytes and hepatocytes, especially those isolated from mice or human donors with obesity and metabolic syndromes [114, 115]. Growing evidence reveals that the free fatty acids (FFA) could enhance the production of FetA in hepatocytes and adipocytes via increasing the activation NF-κB signaling pathway. Furthermore, the serum levels of FetA significantly reduce after the treatment of weight loss in obese patients, indicating consequently that FetA is associated with obesity [116].

On the other hand, the role of FetA in the pathogenesis of inflammation, both in the systemic and tissue-specific inflammation, has received substantial attention in recent years. As described previously, FetA could induce the synthesis and secretion of pro-inflammatory adipokines and synergistically inhibit the lipid efflux especially within macrophages [117, 118]. In addition, the adipocyte-derived FetA shows the effect on the transformation of the anti-inflammatory M2-phenotype macrophages into the pro-inflammatory M1-phenotype macrophages, thus promoting the inflammatory process in humans [116]. These results suggest a potential mechanism by which FetA influences the process of inflammation. Given that psoriasis is a chronic systemic inflammatory disease, we could make a reasonable speculation that FetA could also have a role in the pathogenesis of psoriasis. Nonetheless, few studies have reported the relationship between FetA levels and psoriasis. It is worth noting that Genc et al. found that the FetA levels in psoriatic patients was higher than those in healthy individuals, suggesting that FetA may have a role in psoriasis pathogenesis [119]. Likewise, Uyar et al. also found that serum levels of FetA in psoriatic patients was positively correlated with PASI scores.

However, due to the lack of evidence supporting a direct mechanism whereby FetA affect psoriasis, further efforts should be made to elucidate the important role of FetA in promoting obesity and psoriasis.

#### LCN2

More recently, another adipokine named LCN2 has attracted broad attention. LCN2, expressed in human livers, lungs, kidneys and adipose tissues [120], is a 25 kDa glycoprotein member of the highly heterogeneous family of lipocalins. Previous studies have demonstrated that LCN-2 is a component of the innate immune system with a relevant role in the acute phase response to infection as well in as the induction of apoptosis; concurrently, LCN2 is also shown to involve in several inflammatory diseases, including the epidermal inflammation, the inflammatory bowel disease (IBD) and atherosclerotic diseases.

For instance, Baran et al. found that the serum levels of LCN2 were significantly increased in psoriatic patients compared to the healthy controls [110], and two other independent studies have revealed that the serum levels of LCN2 in psoriaeic patients correlated with PASI scores [112, 121]. Likewise, in an animal study, Hau et al. found that the erythema and scaling skin isolated from IMQ-induced psoriatic mice presented higher expression level of LCN2 gene, suggesting that LCN2 plays an important role in promoting the development of psoriasis [75]. Interestingly, these findings have been replicated in other studies from around the world. Wolk et al. and Hadidi et al. provided the evidence that LCN2 is significantly up-regulated in keratinocytes of psoriatic skin lesions, and the LCN2 level is positively correlated with IL-1β [88, 122], while IL-1β promotes obesity and psoriasis synergistically, thus emphasizing the regulatory nature of LCN2 in linking psoriasis and obesity.

## Treatment of obesity and psoriasis through targeting adipokines

Several studies have shown that the treatment of weight loss in obese patients may effectively improve the pathogenesis of psoriasis. Alternatively, the effects of diet control or exercise in obese patients with moderate-to-severe psoriasis also revealed that the mean weight of these patients was significantly lower in the diet-controlling group compared to that in the control group. In addition, around 66.7% of the obese patients in the diet-controlling group achieved about 75% decrease in their PASI score (PASI-75), and the percentage of patients who achieved PASI-75 was only around 29% in the control group. Mechanically, the authors also found that a low-calorie diet could further reduce the serum level of leptin and simultaneously increased the serum level of adiponectin [123, 124], indicating that lifestyle modifications may be the supplements in addition to the pharmacologic treatment of obese patients with psoriasis.

On the other hand, several studies have already pointed to the regulation of the concentration of adipokines and have demonstrated that the treatment of weight loss in obese patients with psoriasis should focus more on reducing obesity-induced inflammation and adipokine secretion. More recently, studies have signified that the psoriatic patients treated with the fumaric acid esters or methotrexate had an increased serum level of adiponectin compared to that in patients per se before treatment [125–127]. In addition, the psoriatic patients treated with anti-TNFα agents also had a significantly increase in the serum level of adiponectin and a reduction in the serum level of IL-6, suggesting that aiming at adipokines might help to treat psoriasis and obesity [128]. Nevertheless, we still need more large-scale prospective studies in patients to determine the validity of the values about the treating effect.

## Conclusions and perspectives

In summary, there is a complex relationship between obesity, psoriasis and adipokines. According to the results of studies, psoriasis and obesity may not be reciprocally causal but may be derived from a shared pathophysiology. To this point, future studies focusing on the relationship are important, not only from the public health perspective but also to achieve more comprehensive management of psoriasis. Before a decision is made about which the therapeutic intervention should be applied to managing a psoriatic patient, it seems important to consider synchronously that the body weight of the patients is also involved in the management. Psoriatic patients may be evaluated from both a dermatological and a metabolic perspective.

Currently, the PASI is being used to evaluate the severity of psoriasis and the affected body areas; meanwhile, the evaluation of bodyweight would also be valuable and would complement the clinical assessment of the psoriatic patients. The complex interaction of psoriasis with different comorbidities suggests the need for a multidisciplinary approach in the management of obese patients with psoriasis.

Further research is required to elucidate the role of adipokines in patients with psoriasis and psoriasis-related comorbidities. The assessment of the serum levels of adipokines in a well-phenotyped population with psoriasis, while controlling for endocrine factors, is important for further understanding the disease. Adipokines may be mediators of cutaneous inflammation, which suggests a role in the pathogenesis of psoriasis and the development of obesity.

## Data Availability

Not applicable.

## Abbreviations

BMI: Body mass index
CAMP: Cyclic adenosine monophosphate
CCRL2: Chemokine CC-motif receptor-like 2
ERK1/2: Extracellular regulated protein kinases 1 and 2
FetA: Fetuin-A
FFA: Free fatty acids
GPR1G: Protein coupled receptor 1
HMW: High molecular weight
IBD: Inflammatory bowel disease
IL-6: Interleukin-6
LCN2: Lipocalin-2
NF-κB: Nuclear factor kappa B
NO: Nitric oxide
NOS: Nitric oxide synthase
PASI: Psoriasis area and severity index
pDC: Plasmacytoid dendritic cells
PI3K: Phosphatidylinositol-3 kinase
PPARγ: Peroxisome proliferator-activated receptor gamma
RA: Rheumatoid arthritis
RBP4: Retinol binding protein 4
Reg3γ: Regenerating islet-derived 3-gamma
ROR-γt: Retinoid-related orphan receptor-gamma-t
ROS: Reactive oxygen species
SIRT1: Sirtuin-1
TLRs: Toll-like receptors
TNF-α: Tumor necrosis factor-alpha
VEGF: Vascular endothelial growth factors
WHR: Waist-hip ratio
